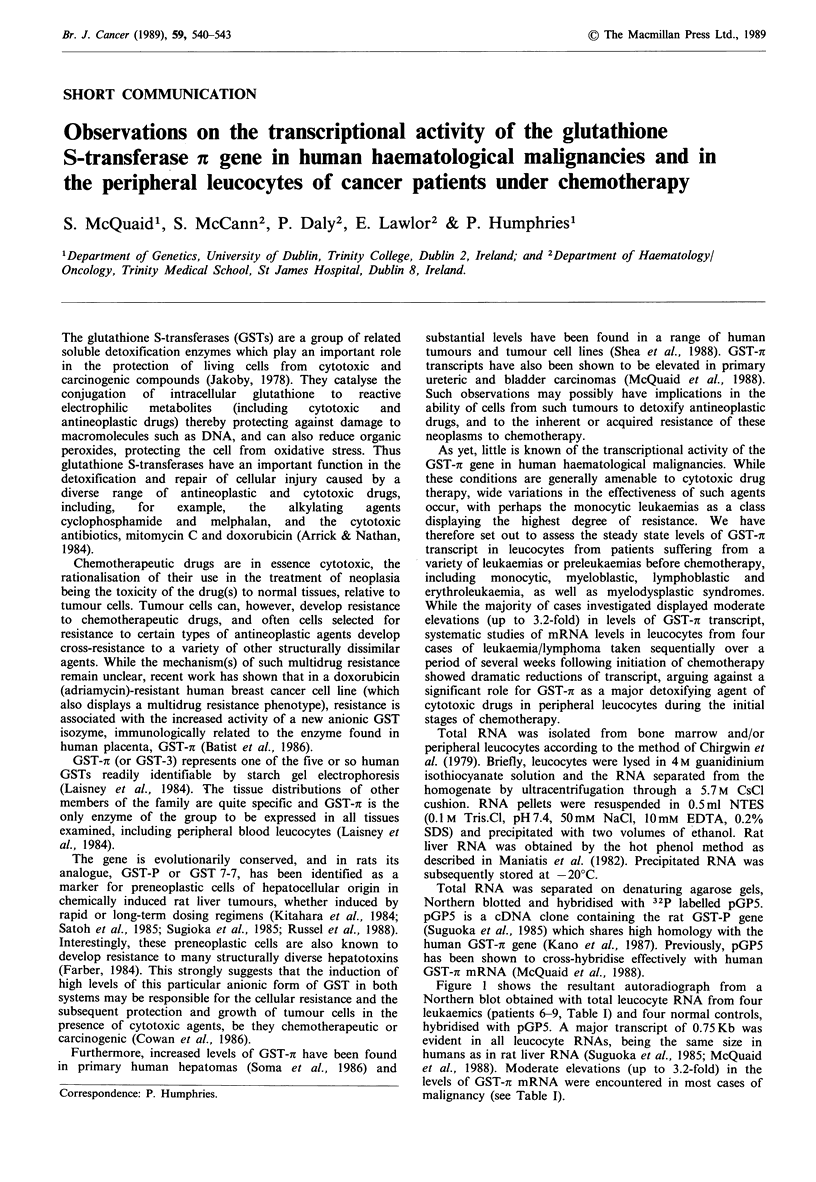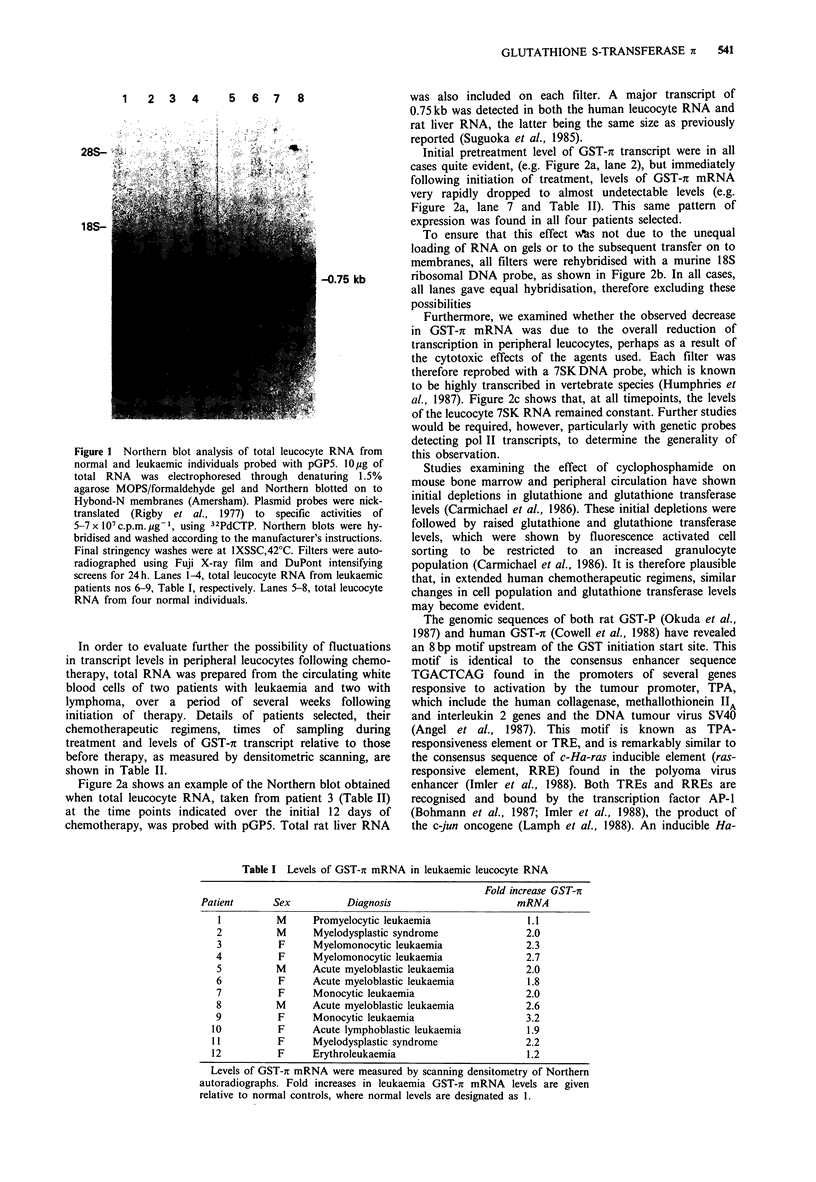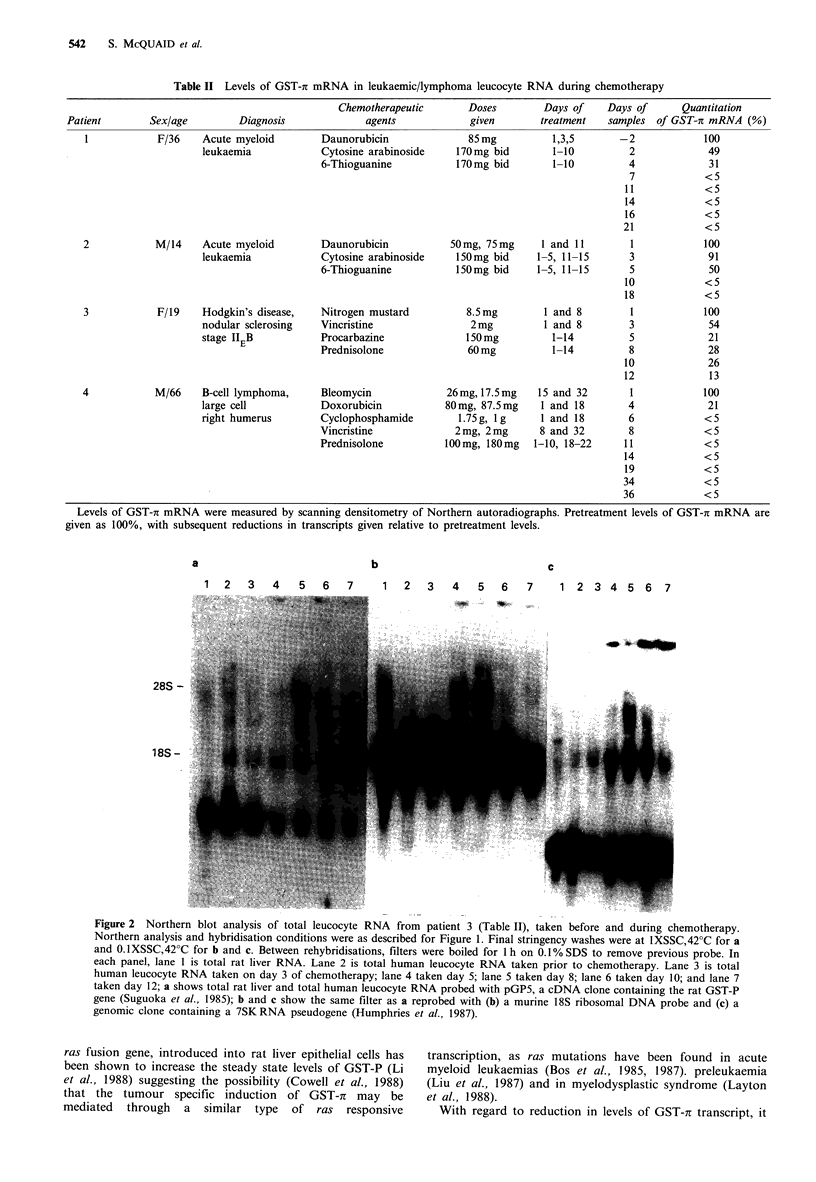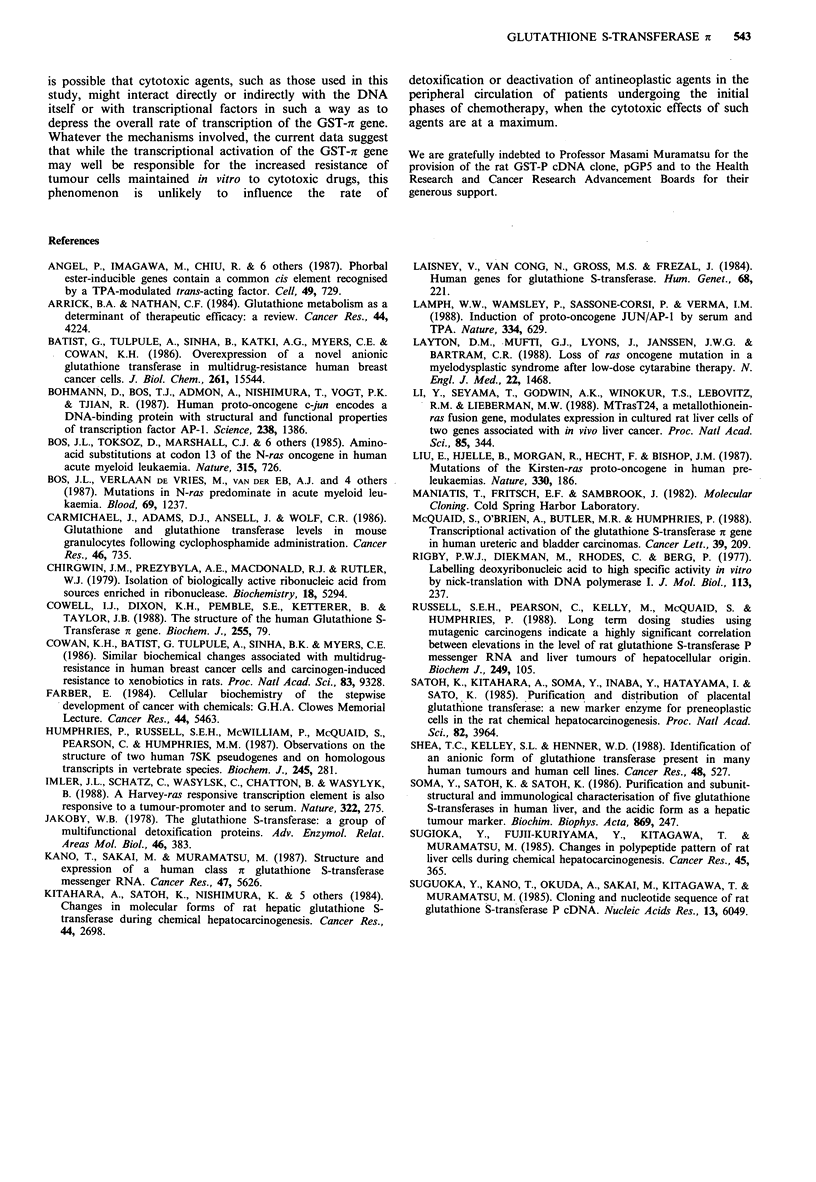# Observations on the transcriptional activity of the glutathione S-transferase pi gene in human haematological malignancies and in the peripheral leucocytes of cancer patients under chemotherapy.

**DOI:** 10.1038/bjc.1989.110

**Published:** 1989-04

**Authors:** S. McQuaid, S. McCann, P. Daly, E. Lawlor, P. Humphries

**Affiliations:** Department of Genetics, University of Dublin, Trinity College, Ireland.

## Abstract

**Images:**


Br. J. Cancer (1989), 59, 540–543 © The Macmillan Press Ltd., 1989

SHORT COMMUNICATION

### Observations on the transcriptional activity of the glutathione S-transferase π gene in human haematological malignancies and in the peripheral leucocytes of cancer patients under chemotherapy

S. McQuaid[1], S. McCann[2], P. Daly[2], E. Lawlor[2] & P. Humphries[1]

[1]*Department of Genetics, University of Dublin, Trinity College, Dublin 2, Ireland; and* [2]*Department of Haematology/Oncology, Trinity Medical School, St James Hospital, Dublin 8, Ireland.*

The glutathione S-transferases (GSTs) are a group of related soluble detoxification enzymes which play an important role in the protection of living cells from cytotoxic and carcinogenic compounds (Jakoby, 1978). They catalyse the conjugation of intracellular glutathione to reactive electrophilic metabolites (including cytotoxic and antineoplastic drugs) thereby protecting against damage to macromolecules such as DNA, and can also reduce organic peroxides, protecting the cell from oxidative stress. Thus glutathione S-transferases have an important function in the detoxification and repair of cellular injury caused by a diverse range of antineoplastic and cytotoxic drugs, including, for example, the alkylating agents cyclophosphamide and melphalan, and the cytotoxic antibiotics, mitomycin C and doxorubicin (Arrick & Nathan, 1984).

Chemotherapeutic drugs are in essence cytotoxic, the rationalisation of their use in the treatment of neoplasia being the toxicity of the drug(s) to normal tissues, relative to tumour cells. Tumour cells can, however, develop resistance to chemotherapeutic drugs, and often cells selected for resistance to certain types of antineoplastic agents develop cross-resistance to a variety of other structurally dissimilar agents. While the mechanism(s) of such multidrug resistance remain unclear, recent work has shown that in a doxorubicin (adriamycin)-resistant human breast cancer cell line (which also displays a multidrug resistance phenotype), resistance is associated with the increased activity of a new anionic GST isozyme, immunologically related to the enzyme found in human placenta, GST-π (Batist *et al.*, 1986).

GST-π (or GST-3) represents one of the five or so human GSTs readily identifiable by starch gel electrophoresis (Laisney *et al.*, 1984). The tissue distributions of other members of the family are quite specific and GST-π is the only enzyme of the group to be expressed in all tissues examined, including peripheral blood leucocytes (Laisney *et al.*, 1984).

The gene is evolutionarily conserved, and in rats its analogue, GST-P or GST 7-7, has been identified as a marker for preneoplastic cells of hepatocellular origin in chemically induced rat liver tumours, whether induced by rapid or long-term dosing regimens (Kitahara *et al.*, 1984; Satoh *et al.*, 1985; Sugioka *et al.*, 1985; Russel *et al.*, 1988). Interestingly, these preneoplastic cells are also known to develop resistance to many structurally diverse hepatotoxins (Farber, 1984). This strongly suggests that the induction of high levels of this particular anionic form of GST in both systems may be responsible for the cellular resistance and the subsequent protection and growth of tumour cells in the presence of cytotoxic agents, be they chemotherapeutic or carcinogenic (Cowan *et al.*, 1986).

Furthermore, increased levels of GST-π have been found in primary human hepatomas (Soma *et al.*, 1986) and substantial levels have been found in a range of human tumours and tumour cell lines (Shea *et al.*, 1988). GST-π transcripts have also been shown to be elevated in primary ureteric and bladder carcinomas (McQuaid *et al.*, 1988). Such observations may possibly have implications in the ability of cells from such tumours to detoxify antineoplastic drugs, and to the inherent or acquired resistance of these neoplasms to chemotherapy.

Correspondence: P. Humphries.

As yet, little is known of the transcriptional activity of the GST-π gene in human haematological malignancies. While these conditions are generally amenable to cytotoxic drug therapy, wide variations in the effectiveness of such agents occur, with perhaps the monocytic leukaemias as a class displaying the highest degree of resistance. We have therefore set out to assess the steady state levels of GST-π transcript in leucocytes from patients suffering from a variety of leukaemias or preleukaemias before chemotherapy, including monocytic, myeloblastic, lymphoblastic and erythroleukaemia, as well as myelodysplastic syndromes. While the majority of cases investigated displayed moderate elevations (up to 3.2-fold) in levels of GST-π transcript, systematic studies of mRNA levels in leucocytes from four cases of leukaemia/lymphoma taken sequentially over a period of several weeks following initiation of chemotherapy showed dramatic reductions of transcript, arguing against a significant role for GST-π as a major detoxifying agent of cytotoxic drugs in peripheral leucocytes during the initial stages of chemotherapy.

Total RNA was isolated from bone marrow and/or peripheral leucocytes according to the method of Chirgwin *et al.* (1979). Briefly, leucocytes were lysed in 4 M guanidinium isothiocyanate solution and the RNA separated from the homogenate by ultracentrifugation through a 5.7 M CsCl cushion. RNA pellets were resuspended in 0.5 ml NTES (0.1 M Tris.Cl, pH 7.4, 50 mM NaCl, 10 mM EDTA, 0.2% SDS) and precipitated with two volumes of ethanol. Rat liver RNA was obtained by the hot phenol method as described in Maniatis *et al.* (1982). Precipitated RNA was subsequently stored at −20°C.

Total RNA was separated on denaturing agarose gels, Northern blotted and hybridised with $^{32}P$ labelled pGP5. pGP5 is a cDNA clone containing the rat GST-P gene (Suguoka *et al.*, 1985) which shares high homology with the human GST-π gene (Kano *et al.*, 1987). Previously, pGP5 has been shown to cross-hybridise effectively with human GST-π mRNA (McQuaid *et al.*, 1988).

Figure 1 shows the resultant autoradiograph from a Northern blot obtained with total leucocyte RNA from four leukaemics (patients 6–9, Table I) and four normal controls, hybridised with pGP5. A major transcript of 0.75 Kb was evident in all leucocyte RNAs, being the same size in humans as in rat liver RNA (Suguoka *et al.*, 1985; McQuaid *et al.*, 1988). Moderate elevations (up to 3.2-fold) in the levels of GST-π mRNA were encountered in most cases of malignancy (see Table I).

**Figure 1** Northern blot analysis of total leucocyte RNA from normal and leukaemic individuals probed with pGP5. 10 μg of total RNA was electrophoresed through denaturing 1.5% agarose MOPS/formaldehyde gel and Northern blotted on to Hybond-N membranes (Amersham). Plasmid probes were nick-translated (Rigby *et al.*, 1977) to specific activities of $5–7 \times 10^7$ c.p.m. $\mu g^{-1}$, using $^{32}$PdCTP. Northern blots were hybridised and washed according to the manufacturer's instructions. Final stringency washes were at 1XSSC,42°C. Filters were autoradiographed using Fuji X-ray film and DuPont intensifying screens for 24 h. Lanes 1–4, total leucocyte RNA from leukaemic patients nos 6–9, Table I, respectively. Lanes 5–8, total leucocyte RNA from four normal individuals.

In order to evaluate further the possibility of fluctuations in transcript levels in peripheral leucocytes following chemotherapy, total RNA was prepared from the circulating white blood cells of two patients with leukaemia and two with lymphoma, over a period of several weeks following initiation of therapy. Details of patients selected, their chemotherapeutic regimens, times of sampling during treatment and levels of GST-π transcript relative to those before therapy, as measured by densitometric scanning, are shown in Table II.

Figure 2a shows an example of the Northern blot obtained when total leucocyte RNA, taken from patient 3 (Table II) at the time points indicated over the initial 12 days of chemotherapy, was probed with pGP5. Total rat liver RNA was also included on each filter. A major transcript of 0.75 kb was detected in both the human leucocyte RNA and rat liver RNA, the latter being the same size as previously reported (Suguoka *et al.*, 1985).

Initial pretreatment level of GST-π transcript were in all cases quite evident, (e.g. Figure 2a, lane 2), but immediately following initiation of treatment, levels of GST-π mRNA very rapidly dropped to almost undetectable levels (e.g. Figure 2a, lane 7 and Table II). This same pattern of expression was found in all four patients selected.

To ensure that this effect was not due to the unequal loading of RNA on gels or to the subsequent transfer on to membranes, all filters were rehybridised with a murine 18S ribosomal DNA probe, as shown in Figure 2b. In all cases, all lanes gave equal hybridisation, therefore excluding these possibilities

Furthermore, we examined whether the observed decrease in GST-π mRNA was due to the overall reduction of transcription in peripheral leucocytes, perhaps as a result of the cytotoxic effects of the agents used. Each filter was therefore reprobed with a 7SK DNA probe, which is known to be highly transcribed in vertebrate species (Humphries *et al.*, 1987). Figure 2c shows that, at all timepoints, the levels of the leucocyte 7SK RNA remained constant. Further studies would be required, however, particularly with genetic probes detecting pol II transcripts, to determine the generality of this observation.

Studies examining the effect of cyclophosphamide on mouse bone marrow and peripheral circulation have shown initial depletions in glutathione and glutathione transferase levels (Carmichael *et al.*, 1986). These initial depletions were followed by raised glutathione and glutathione transferase levels, which were shown by fluorescence activated cell sorting to be restricted to an increased granulocyte population (Carmichael *et al.*, 1986). It is therefore plausible that, in extended human chemotherapeutic regimens, similar changes in cell population and glutathione transferase levels may become evident.

The genomic sequences of both rat GST-P (Okuda *et al.*, 1987) and human GST-π (Cowell *et al.*, 1988) have revealed an 8 bp motif upstream of the GST initiation start site. This motif is identical to the consensus enhancer sequence TGACTCAG found in the promoters of several genes responsive to activation by the tumour promoter, TPA, which include the human collagenase, methallothionein $II_A$ and interleukin 2 genes and the DNA tumour virus SV40 (Angel *et al.*, 1987). This motif is known as TPA-responsiveness element or TRE, and is remarkably similar to the consensus sequence of *c-Ha-ras* inducible element (*ras*-responsive element, RRE) found in the polyoma virus enhancer (Imler *et al.*, 1988). Both TREs and RREs are recognised and bound by the transcription factor AP-1 (Bohmann *et al.*, 1987; Imler *et al.*, 1988), the product of the c-*jun* oncogene (Lamph *et al.*, 1988). An inducible *Ha-*

**Table I** Levels of GST-π mRNA in leukaemic leucocyte RNA

| *Patient* | *Sex* | *Diagnosis* | *Fold increase GST-π mRNA* |
|---|---|---|---|
| 1 | M | Promyelocytic leukaemia | 1.1 |
| 2 | M | Myelodysplastic syndrome | 2.0 |
| 3 | F | Myelomonocytic leukaemia | 2.3 |
| 4 | F | Myelomonocytic leukaemia | 2.7 |
| 5 | M | Acute myeloblastic leukaemia | 2.0 |
| 6 | F | Acute myeloblastic leukaemia | 1.8 |
| 7 | F | Monocytic leukaemia | 2.0 |
| 8 | M | Acute myeloblastic leukaemia | 2.6 |
| 9 | F | Monocytic leukaemia | 3.2 |
| 10 | F | Acute lymphoblastic leukaemia | 1.9 |
| 11 | F | Myelodysplastic syndrome | 2.2 |
| 12 | F | Erythroleukaemia | 1.2 |

Levels of GST-π mRNA were measured by scanning densitometry of Northern autoradiographs. Fold increases in leukaemia GST-π mRNA levels are given relative to normal controls, where normal levels are designated as 1.

**Table II** Levels of GST-π mRNA in leukaemic/lymphoma leucocyte RNA during chemotherapy

| *Patient* | *Sex/age* | *Diagnosis* | *Chemotherapeutic agents* | *Doses given* | *Days of treatment* | *Days of samples* | *Quantitation of GST-π mRNA (%)* |
|---|---|---|---|---|---|---|---|
| 1 | F/36 | Acute myeloid leukaemia | Daunorubicin | 85 mg | 1,3,5 | −2 | 100 |
| | | | Cytosine arabinoside | 170 mg bid | 1–10 | 2 | 49 |
| | | | 6-Thioguanine | 170 mg bid | 1–10 | 4 | 31 |
| | | | | | | 7 | <5 |
| | | | | | | 11 | <5 |
| | | | | | | 14 | <5 |
| | | | | | | 16 | <5 |
| | | | | | | 21 | <5 |
| 2 | M/14 | Acute myeloid leukaemia | Daunorubicin | 50 mg, 75 mg | 1 and 11 | 1 | 100 |
| | | | Cytosine arabinoside | 150 mg bid | 1–5, 11–15 | 3 | 91 |
| | | | 6-Thioguanine | 150 mg bid | 1–5, 11–15 | 5 | 50 |
| | | | | | | 10 | <5 |
| | | | | | | 18 | <5 |
| 3 | F/19 | Hodgkin's disease, nodular sclerosing stage $II_EB$ | Nitrogen mustard | 8.5 mg | 1 and 8 | 1 | 100 |
| | | | Vincristine | 2 mg | 1 and 8 | 3 | 54 |
| | | | Procarbazine | 150 mg | 1–14 | 5 | 21 |
| | | | Prednisolone | 60 mg | 1–14 | 8 | 28 |
| | | | | | | 10 | 26 |
| | | | | | | 12 | 13 |
| 4 | M/66 | B-cell lymphoma, large cell right humerus | Bleomycin | 26 mg, 17.5 mg | 15 and 32 | 1 | 100 |
| | | | Doxorubicin | 80 mg, 87.5 mg | 1 and 18 | 4 | 21 |
| | | | Cyclophosphamide | 1.75 g, 1 g | 1 and 18 | 6 | <5 |
| | | | Vincristine | 2 mg, 2 mg | 8 and 32 | 8 | <5 |
| | | | Prednisolone | 100 mg, 180 mg | 1–10, 18–22 | 11 | <5 |
| | | | | | | 14 | <5 |
| | | | | | | 19 | <5 |
| | | | | | | 34 | <5 |
| | | | | | | 36 | <5 |

Levels of GST-π mRNA were measured by scanning densitometry of Northern autoradiographs. Pretreatment levels of GST-π mRNA are given as 100%, with subsequent reductions in transcripts given relative to pretreatment levels.

**Figure 2** Northern blot analysis of total leucocyte RNA from patient 3 (Table II), taken before and during chemotherapy. Northern analysis and hybridisation conditions were as described for Figure 1. Final stringency washes were at 1XSSC,42°C for **a** and 0.1XSSC,42°C for **b** and **c**. Between rehybridisations, filters were boiled for 1 h on 0.1% SDS to remove previous probe. In each panel, lane 1 is total rat liver RNA. Lane 2 is total human leucocyte RNA taken prior to chemotherapy. Lane 3 is total human leucocyte RNA taken on day 3 of chemotherapy; lane 4 taken day 5; lane 5 taken day 8; lane 6 taken day 10; and lane 7 taken day 12; **a** shows total rat liver and total human leucocyte RNA probed with pGP5, a cDNA clone containing the rat GST-P gene (Suguoka *et al.*, 1985); **b** and **c** show the same filter as **a** reprobed with (**b**) a murine 18S ribosomal DNA probe and (**c**) a genomic clone containing a 7SK RNA pseudogene (Humphries *et al.*, 1987).

*ras* fusion gene, introduced into rat liver epithelial cells has been shown to increase the steady state levels of GST-P (Li *et al.*, 1988) suggesting the possibility (Cowell *et al.*, 1988) that the tumour specific induction of GST-π may be mediated through a similar type of *ras* responsive transcription, as *ras* mutations have been found in acute myeloid leukaemias (Bos *et al.*, 1985, 1987). preleukaemia (Liu *et al.*, 1987) and in myelodysplastic syndrome (Layton *et al.*, 1988).

With regard to reduction in levels of GST-π transcript, it is possible that cytotoxic agents, such as those used in this study, might interact directly or indirectly with the DNA itself or with transcriptional factors in such a way as to depress the overall rate of transcription of the GST-π gene. Whatever the mechanisms involved, the current data suggest that while the transcriptional activation of the GST-π gene may well be responsible for the increased resistance of tumour cells maintained *in vitro* to cytotoxic drugs, this phenomenon is unlikely to influence the rate of detoxification or deactivation of antineoplastic agents in the peripheral circulation of patients undergoing the initial phases of chemotherapy, when the cytotoxic effects of such agents are at a maximum.

We are gratefully indebted to Professor Masami Muramatsu for the provision of the rat GST-P cDNA clone, pGP5 and to the Health Research and Cancer Research Advancement Boards for their generous support.

### References

ANGEL, P., IMAGAWA, M., CHIU, R. & 6 others (1987). Phorbal ester-inducible genes contain a common *cis* element recognised by a TPA-modulated *trans*-acting factor. *Cell*, **49**, 729.

ARRICK, B.A. & NATHAN, C.F. (1984). Glutathione metabolism as a determinant of therapeutic efficacy: a review. *Cancer Res.*, **44**, 4224.

BATIST, G., TULPULE, A., SINHA, B., KATKI, A.G., MYERS, C.E. & COWAN, K.H. (1986). Overexpression of a novel anionic glutathione transferase in multidrug-resistance human breast cancer cells. *J. Biol. Chem.*, **261**, 15544.

BOHMANN, D., BOS, T.J., ADMON, A., NISHIMURA, T., VOGT, P.K. & TJIAN, R. (1987). Human proto-oncogene c-*jun* encodes a DNA-binding protein with structural and functional properties of transcription factor AP-1. *Science*, **238**, 1386.

BOS, J.L., TOKSOZ, D., MARSHALL, C.J. & 6 others (1985). Amino-acid substitutions at codon 13 of the N-*ras* oncogene in human acute myeloid leukaemia. *Nature*, **315**, 726.

BOS, J.L., VERLAAN DE VRIES, M., VAN DER EB, A.J. and 4 others (1987). Mutations in N-*ras* predominate in acute myeloid leukaemia. *Blood*, **69**, 1237.

CARMICHAEL, J., ADAMS, D.J., ANSELL, J. & WOLF, C.R. (1986). Glutathione and glutathione transferase levels in mouse granulocytes following cyclophosphamide administration. *Cancer Res.*, **46**, 735.

CHIRGWIN, J.M., PREZYBYLA, A.E., MACDONALD, R.J. & RUTLER, W.J. (1979). Isolation of biologically active ribonucleic acid from sources enriched in ribonuclease. *Biochemistry*, **18**, 5294.

COWELL, I.J., DIXON, K.H., PEMBLE, S.E., KETTERER, B. & TAYLOR, J.B. (1988). The structure of the human Glutathione S-Transferase π gene. *Biochem. J.*, **255**, 79.

COWAN, K.H., BATIST, G. TULPULE, A., SINHA, B.K. & MYERS, C.E. (1986). Similar biochemical changes associated with multidrug-resistance in human breast cancer cells and carcinogen-induced resistance to xenobiotics in rats. *Proc. Natl Acad. Sci.*, **83**, 9328.

FARBER, E. (1984). Cellular biochemistry of the stepwise development of cancer with chemicals: G.H.A. Clowes Memorial Lecture. *Cancer Res.*, **44**, 5463.

HUMPHRIES, P., RUSSELL, S.E.H., McWILLIAM, P., McQUAID, S., PEARSON, C. & HUMPHRIES, M.M. (1987). Observations on the structure of two human 7SK pseudogenes and on homologous transcripts in vertebrate species. *Biochem. J.*, **245**, 281.

IMLER, J.L., SCHATZ, C., WASYLSK, C., CHATTON, B. & WASYLYK, B. (1988). A Harvey-*ras* responsive transcription element is also responsive to a tumour-promoter and to serum. *Nature*, **322**, 275.

JAKOBY, W.B. (1978). The glutathione S-transferase: a group of multifunctional detoxification proteins. *Adv. Enzymol. Relat. Areas Mol. Biol.*, **46**, 383.

KANO, T., SAKAI, M. & MURAMATSU, M. (1987). Structure and expression of a human class π glutathione S-transferase messenger RNA. *Cancer Res.*, **47**, 5626.

KITAHARA, A., SATOH, K., NISHIMURA, K. & 5 others (1984). Changes in molecular forms of rat hepatic glutathione S-transferase during chemical hepatocarcinogenesis. *Cancer Res.*, **44**, 2698.

LAISNEY, V., VAN CONG, N., GROSS, M.S. & FREZAL, J. (1984). Human genes for glutathione S-transferase. *Hum. Genet.*, **68**, 221.

LAMPH, W.W., WAMSLEY, P., SASSONE-CORSI, P. & VERMA, I.M. (1988). Induction of proto-oncogene JUN/AP-1 by serum and TPA. *Nature*, **334**, 629.

LAYTON, D.M., MUFTI, G.J., LYONS, J., JANSSEN, J.W.G. & BARTRAM, C.R. (1988). Loss of *ras* oncogene mutation in a myelodysplastic syndrome after low-dose cytarabine therapy. *N. Engl. J. Med.*, **22**, 1468.

LI, Y., SEYAMA, T., GODWIN, A.K., WINOKUR, T.S., LEBOVITZ, R.M. & LIEBERMAN, M.W. (1988). MTrasT24, a metallothionein-*ras* fusion gene, modulates expression in cultured rat liver cells of two genes associated with *in vivo* liver cancer. *Proc. Natl Acad. Sci.*, **85**, 344.

LIU, E., HJELLE, B., MORGAN, R., HECHT, F. & BISHOP, J.M. (1987). Mutations of the Kirsten-*ras* proto-oncogene in human pre-leukaemias. *Nature*, **330**, 186.

MANIATIS, T., FRITSCH, E.F. & SAMBROOK, J. (1982). *Molecular Cloning*. Cold Spring Harbor Laboratory.

McQUAID, S., O'BRIEN, A., BUTLER, M.R. & HUMPHRIES, P. (1988). Transcriptional activation of the glutathione S-transferase π gene in human ureteric and bladder carcinomas. *Cancer Lett.*, **39**, 209.

RIGBY, P.W.J., DIEKMAN, M., RHODES, C. & BERG, P. (1977). Labelling deoxyribonucleic acid to high specific activity *in vitro* by nick-translation with DNA polymerase I. *J. Mol. Biol.*, **113**, 237.

RUSSELL, S.E.H., PEARSON, C., KELLY, M., McQUAID, S. & HUMPHRIES, P. (1988). Long term dosing studies using mutagenic carcinogens indicate a highly significant correlation between elevations in the level of rat glutathione S-transferase P messenger RNA and liver tumours of hepatocellular origin. *Biochem J.*, **249**, 105.

SATOH, K., KITAHARA, A., SOMA, Y., INABA, Y., HATAYAMA, I. & SATO, K. (1985). Purification and distribution of placental glutathione transferase: a new marker enzyme for preneoplastic cells in the rat chemical hepatocarcinogenesis. *Proc. Natl Acad. Sci.*, **82**, 3964.

SHEA, T.C., KELLEY, S.L. & HENNER, W.D. (1988). Identification of an anionic form of glutathione transferase present in many human tumours and human cell lines. *Cancer Res.*, **48**, 527.

SOMA, Y., SATOH, K. & SATOH, K. (1986). Purification and subunit-structural and immunological characterisation of five glutathione S-transferases in human liver, and the acidic form as a hepatic tumour marker. *Biochim. Biophys. Acta*, **869**, 247.

SUGIOKA, Y., FUJII-KURIYAMA, Y., KITAGAWA, T. & MURAMATSU, M. (1985). Changes in polypeptide pattern of rat liver cells during chemical hepatocarcinogenesis. *Cancer Res.*, **45**, 365.

SUGUOKA, Y., KANO, T., OKUDA, A., SAKAI, M., KITAGAWA, T. & MURAMATSU, M. (1985). Cloning and nucleotide sequence of rat glutathione S-transferase P cDNA. *Nucleic Acids Res.*, **13**, 6049.